# Exploring the role of APRIL in autoimmunity: implications for therapeutic targeting in systemic lupus erythematosus, rheumatoid arthritis, and Sjögren’s syndrome

**DOI:** 10.3389/fimmu.2025.1523392

**Published:** 2025-08-01

**Authors:** Anastasia V. Poznyak, Elena V. Gerasimova, Nikolay A. Orekhov, Amina Eldarovna Karimova, Maria Andreevna Vergun, Ksenia Olegovna Lapshina, Vasily N. Sukhorukov, Alexander N. Orekhov

**Affiliations:** ^1^ Institute for Atherosclerosis Research, Moscow, Russia; ^2^ Department of Systemic Rheumatic Diseases, V.A. Nasonova Research Institute of Rheumatology, Moscow, Russia; ^3^ Laboratory of Angiopathology, Institute of General Pathology and Pathophysiology, Moscow, Russia; ^4^ Faculty of Biology and Biotechnology, National Research University Higher School of Economics, Moscow, Russia

**Keywords:** APRIL (TNFSF13), BAFF, B cells, autoimmunity, rheumatoid arthritis

## Abstract

Autoimmunity arises when the immune system erroneously attacks self-antigens, potentially resulting in organ dysfunction. This review focuses on the proliferation-inducing ligand, APRIL, and its critical role in regulating antibody-producing B cells. We explore the implications of APRIL in autoimmune diseases such as systemic lupus erythematosus, rheumatoid arthritis, and Sjögren’s syndrome. Emerging evidence indicates that APRIL may modulate autoimmune pathology and influence B cell survival, particularly through its interactions with receptors like B-cell maturation antigen (BCMA) and transmembrane activator and CAML interactor (TACI). We emphasize the contrasting roles of APRIL and BAFF in autoimmunity, highlighting the conflicting data regarding their contributions to disease progression and activity levels. Furthermore, we evaluate therapeutic strategies aimed at inhibiting APRIL and compare them with existing B-cell-targeted therapies, such as rituximab and belimumab. The potential benefits of specific APRIL antagonism are discussed, especially for patients with antibody-driven autoimmune disorders. This highlights the necessity for further research into APRIL-targeted therapies in clinical practice. Ultimately, this review seeks to provide a comprehensive overview of the current understanding of APRIL’s role in autoimmunity and outline future directions for targeting this ligand in the treatment of autoimmune diseases.

## Introduction

Autoimmunity happens when the adaptive immune system mistakenly targets a self-antigen. In advanced stages, this immune response can extend to multiple self-antigens through a mechanism called epitope spreading. Unfavorably, autoimmunity often results in serious diseases marked by the collapse of the affected organ. Autoantibodies can play a central role in the disease process, initiating cell death that eventually leads to organ dysfunction (1–3). It has long been suggested that molecular mimicry between a self-antigen and a non-self-antigen from an external source, often microbial, could underlie this process. However, during the second Swiss autoimmune liver disease meeting in Lugano, Switzerland, a different concept called dual reactivity was introduced. Dual reactivity also involves an immune response against both self and non-self-antigens, but in this case, the two antigens may have entirely dissimilar structures (4–6). Here, self-reactivity arises due to somatic hypermutations that occur during the germinal center reaction, whereas the original, unmutated ancestor was not autoimmune. This process has been clearly illustrated in pulmonary alveolar proteinosis, where pathogenic autoantibodies are produced against a differentiation factor for alveolar macrophages, known as granulocyte/macrophage colony-stimulating factor (7–9).

This autoimmune reaction results in the depletion of lung macrophages and the agglomeration of harmful substances. Pathogenic autoantibodies may not be the primary cause of the disease but often develop as a secondary response to the initial harm. These antibodies are thought to intensify tissue inflammation and contribute to the intensification of the disease. The strongest evidence for such secondary autoantibodies comes from those targeting cytoplasmic and/or nuclear autoantigens in autoimmune patients (10–12). Given their location within cells, it is evident that the immune system would only encounter these antigens following a primary event, whether autoimmune or not, that leads to cell death. Autoimmune diseases in which molecular mimicry or dual reactivity has been clearly demonstrated are relatively rare. In most instances, the initial steps in the production of both primary and secondary autoantibodies remain unclear, complicating the development of preventive strategies for these antibody-associated autoimmune reactions (13–15). Consequently, therapeutic approaches after disease onset are considered more feasible. Targeting the cells responsible for producing autoantibodies could be particularly effective. There are two well-defined types of antibody-producing cells corresponding to the later stages of B lymphocyte differentiation: plasmablasts (PB), which are formed in secondary lymphoid organs, and fully differentiated plasma cells (PC), which originate from PBs. Antibody-secreting cells can also be produced in non-lymphoid tissues, when ectopic germinal centers are formed (16). Usually, PCs migrate to and settle in the bone marrow, but they can also accumulate in inflamed tissues, such as those affected by autoimmune reactions (17, 18). Unlike PBs, PCs can be extremely long-lived, particularly in the bone marrow, and they persist in tissues until the inflammation subsides. This review will explore the literature on the physiological role of a proliferation-inducing ligand (APRIL) in antibody-producing cells, discuss current insights into APRIL’s role in autoimmune diseases, and consider the potential outcomes of targeting APRIL in comparison to existing B-cell modulating therapies (19–21).

## APRIL and BAFF

APRIL and BAFF (B-cell activating factor) are two critical cytokines that play essential roles in the regulation of B cell survival, maturation, and antibody production. Both cytokines belong to the tumor necrosis factor (TNF) superfamily and interact with specific receptors on B cells to modulate immune responses. BAFF primarily promotes the survival and differentiation of B cells, while APRIL has a unique ability to enhance the survival of long-lived plasma cells and induce antibody production, particularly IgA. Despite their complementary functions, emerging evidence suggests that their roles in autoimmunity can be quite contrasting, with BAFF more commonly associated with promoting pathogenic B cell activity, while APRIL may exert regulatory effects. This complexity prompts further investigation into the therapeutic targeting of these cytokines, as manipulating their balance could provide novel approaches for treating various autoimmune disorders. Understanding the specific roles of APRIL and BAFF in B cell biology is crucial for developing targeted therapies aimed at modulating autoimmunity and improving patient outcomes (20–23).

APRIL uniquely regulates PCs through its affinity for the receptors BCMA (B-cell maturation antigen) and TACI (Transmembrane Activator and CAML-interactor), distinguishing its roles from those of BAFF (B-cell Activating Factor). While both cytokines contribute to the survival and functionality of PCs, APRIL’s interaction with BCMA is especially crucial, as it binds to this receptor with higher affinity, promoting the survival of memory plasma cells predominantly within the bone marrow microenvironment (24). This process significantly activates the NF-κB signaling pathway, which is essential for the maintenance of PCs. Specifically, BCMA signaling helps to upregulate anti-apoptotic proteins like Mcl-1, contributing to cellular resilience. On the other hand, TACI, which is also expressed by plasma cells, assists in inducing the expression of Blimp-1, a transcription factor crucial for maintaining plasma cell identity (25). This dual requirement for both BCMA and TACI signaling illustrates the redundant and complementary roles these receptors play in plasma cell survival by activating critical survival pathways (24).

APRIL specifically supports long-lived plasma cells (LLPCs) through its unique binding affinities and signaling pathways that are distinct from those influencing earlier B cell subsets. Unlike earlier stages of B cell development, which may rely on other factors for maturation, LLPCs predominantly express BCMA and TACI receptors, which are responsive to APRIL (26). When APRIL binds to BCMA, it triggers robust survival signals through the activation of NF-κB pathways, enhancing the longevity of these cells in the bone marrow. Importantly, this signaling also involves the modulation of apoptotic pathways, whereby BCMA signaling upregulates anti-apoptotic proteins like Mcl-1, while TACI contributes to promoting Blimp-1, vital for maintaining plasma cell identity (27). In contrast, earlier B cell subsets do not express these receptors at the same levels, resulting in a lack of the specific support that APRIL provides to LLPCs. This selective signaling ensures that while earlier B cell forms undergo proliferation and selection, only those that have differentiated into plasma cells receive the critical survival signals needed for sustained presence and antibody production in tissues (28).

BAFF significantly influences survival signals during the plasmablast stage through its interaction with specific receptors, particularly BAFFR. Plasmablasts, which are the immediate precursors to plasma cells, rely on BAFF for their survival and maturation. Once BAFF binds to its receptor, it initiates critical intracellular signaling cascades, primarily involving the activation of NF-κB and PI3K pathways (29). These pathways play a crucial role in promoting cell vitality, by enhancing protein synthesis and reducing apoptotic signaling. Additionally, BAFFR activation contributes to the downregulation of pro-apoptotic factors, providing the necessary signals for plasmablasts to thrive in competitive environments such as germinal centers and in the presence of various inflammatory stimuli. Therefore, BAFF acts as a key factor that ensures plasmablasts effectively transition into long-lived plasma cells, supporting the overall humoral immune response (30).

### Specific mechanisms underlying BAFF’s effect

BAFF is instrumental in promoting the survival of immature B cells in the bone marrow. Upon binding to its receptors, particularly BAFF-R, BAFF initiates signaling cascades that enhance the expression of anti-apoptotic proteins, such as Bcl-2. This process is crucial for supporting the survival of immature B lymphocytes during their development.

Following the selection process in the bone marrow, immature B cells that express BAFF-R are positively selected for survival in response to BAFF signaling. This selective survival mechanism ensures that only B cells capable of recognizing self-antigens in a non-autoreactive manner progress toward maturation. When immature B cells express the BAFF receptor (BAFFR), they receive essential pro-survival signals that protect them from apoptosis, allowing for the selection of non-autoreactive B cells. This process helps ensure that only B cells with appropriate reactivity to antigens mature and enter the circulation, contributing to a diverse and functional B cell repertoire. In addition to promoting survival during early development, BAFF also supports the vitality of mature B cells in peripheral lymphoid organs, thus maintaining the homeostasis of the B cell population. This influence extends to enhancing B cell functionality as well; BAFF promotes processes like immunoglobulin class switching and antibody production, essential for effective immune responses (31).

In the peripheral immune system, BAFF is essential for the maintenance and survival of mature B cells, particularly during the transitional and naive stages. Mature B cells rely on BAFF signaling to prevent apoptosis and to prolong their lifespan in circulation, utilizing similar mechanisms that involve anti-apoptotic proteins.

BAFF enhances the ability of B cells to proliferate and differentiate into plasma cells upon activation. BAFF-R signaling is integral to B cell responses to antigens, facilitating processes such as class switching and the generation of high-affinity antibodies, ultimately contributing to effective immune responses.

The binding of BAFF to BAFF-R activates several downstream signaling pathways critical for B cell functions, including:

- NF-κB Pathway: This pathway is vital for B cell survival and proliferation and is activated through IκB kinase (IKK) complex signaling.
- MAPK Pathway: This pathway modulates cellular responses to BAFF and is involved in regulating cell growth and differentiation.
- PI3K-AKT Pathway: This pathway promotes cell survival by inhibiting apoptotic mechanisms.

The binding of BAFF to the BAFF receptor activates several intracellular signaling cascades that promote B cell survival. Among these, the non-canonical NF-κB pathway is crucial; it regulates the transcription of target genes essential for cell survival and proliferation. When BAFF binds to BAFFR, it recruits TRAF3, which leads to the stabilization of NIK, an important kinase (32). As a result, the NF-κB2 pathway is activated, allowing specific transcription factors to enter the nucleus and promote the expression of anti-apoptotic genes like Mcl-1, which is crucial for stabilizing mitochondrial function and extending cell lifespan. Furthermore, BAFFR activation also engages the phosphoinositide-3-kinase (PI3K) signaling pathway, which collaborates with B cell receptor signaling to enhance protein synthesis and improve cellular metabolism (33). Together, these signaling mechanisms ensure that immature B cells avoid premature death and that mature B cells maintain their energy levels and functional capabilities, thus sustaining a robust immune response and balancing the B cell population in the body (34).

Dysregulation of BAFF levels can lead to various clinical conditions, including autoimmune diseases such as systemic lupus erythematosus, characterized by excessive B cell survival and proliferation. Additionally, alterations in BAFF levels can affect the immune response to infections and the efficacy of vaccines.

In summary, BAFF is integral to the survival, maturation, and functionality of B cells, with BAFF-R signaling playing a pivotal role in regulating the pathways that govern B cell homeostasis. Understanding these mechanisms is essential for exploring potential therapeutic targets in the treatment of autoimmune diseases and enhancing immune responses.

## APRIL receptors and signaling

Both B-cell maturation antigen (BCMA) and transmembrane activator and CAML interactor (TACI) are type II transmembrane proteins that lack a signal peptide. Structurally, they resemble other TNFRs, featuring characteristic cysteine-rich domains (CRDs) in their extracellular regions. TACI has two CRDs: CRD2 is responsible for ligand binding, while CRD1 has been identified as forming a pre-ligand assembly domain (PLAD), a feature first described for Fas and TNFR1 (22, 23, 35, 36). The PLAD holds the receptor in a pre-assembled state, ready to interact with an incoming ligand. BCMA is a smaller receptor with only one CRD, which also binds ligands. However, due to the single CRD, it is unlikely to form a PLAD, though this has not been studied in detail. APRIL has been crystallized both as a soluble ligand and in complex with BCMA and the CRD2 (ligand-binding domain) of TACI. These structures have shown that the receptor binding site is located at the C-terminal end of each APRIL in the trimer, leaving the heparane sulfate proteoglycans (HSPGs) domain unoccupied by the receptor (37, 38). HSPGs are large glycoproteins found on the surface of many cells, characterized by covalently attached heparan sulfate chains (39). They play significant roles in cell signaling, cellular adhesion, and the regulation of various biological processes. In the context of APRIL signaling, HSPGs serve as co-receptors that can enhance the interaction between APRIL and its primary receptors, BCMA and TACI. The presence of HSPGs facilitates the clustering of these receptors, which is crucial for effective downstream signaling and activation of B-cell survival pathways (40, 41).

Contrary to other TNFR-ligand complexes, such as DR5 and TRAIL, where receptor monomers bind at the interface between monomers in the trimer, TACI and BCMA receptors bind directly to a single APRIL strand within the trimer (42, 43).

The junction of BCMA and TACI triggers the downstream activation of the classical NF-κB pathway, which is believed to be central to the proliferative signals that harmful B-cells receive. Signaling through BCMA and TACI involves the recruitment of intracellular adaptors known as TNF-receptor associated factors (TRAFs). Initially, TACI was identified as a receptor interacting with CAML, capable of signaling via the NFAT/AP-1 pathway (44–47). However, it was later discovered that TACI can also signal the activation of NF-κB and c-Jun NH2-terminal Kinase (JNK). Through yeast two-hybrid assays, the intracellular domain of TACI was found to join TRAF-2, -5, and -6, and it was determined that the TRAF- and CAML-binding sites are separate. BCMA has also been demonstrated to activate p38 mitogen-activated protein kinase (MAPK) and c-Jun NH2-terminal Kinase (JNK). Nevertheless, the intricate details of complex formation following receptor joining are not well understood for either receptor, and the specific signaling components required to produce distinct signals through the same receptor remain unclear (48, 49).

**Figure 1:**
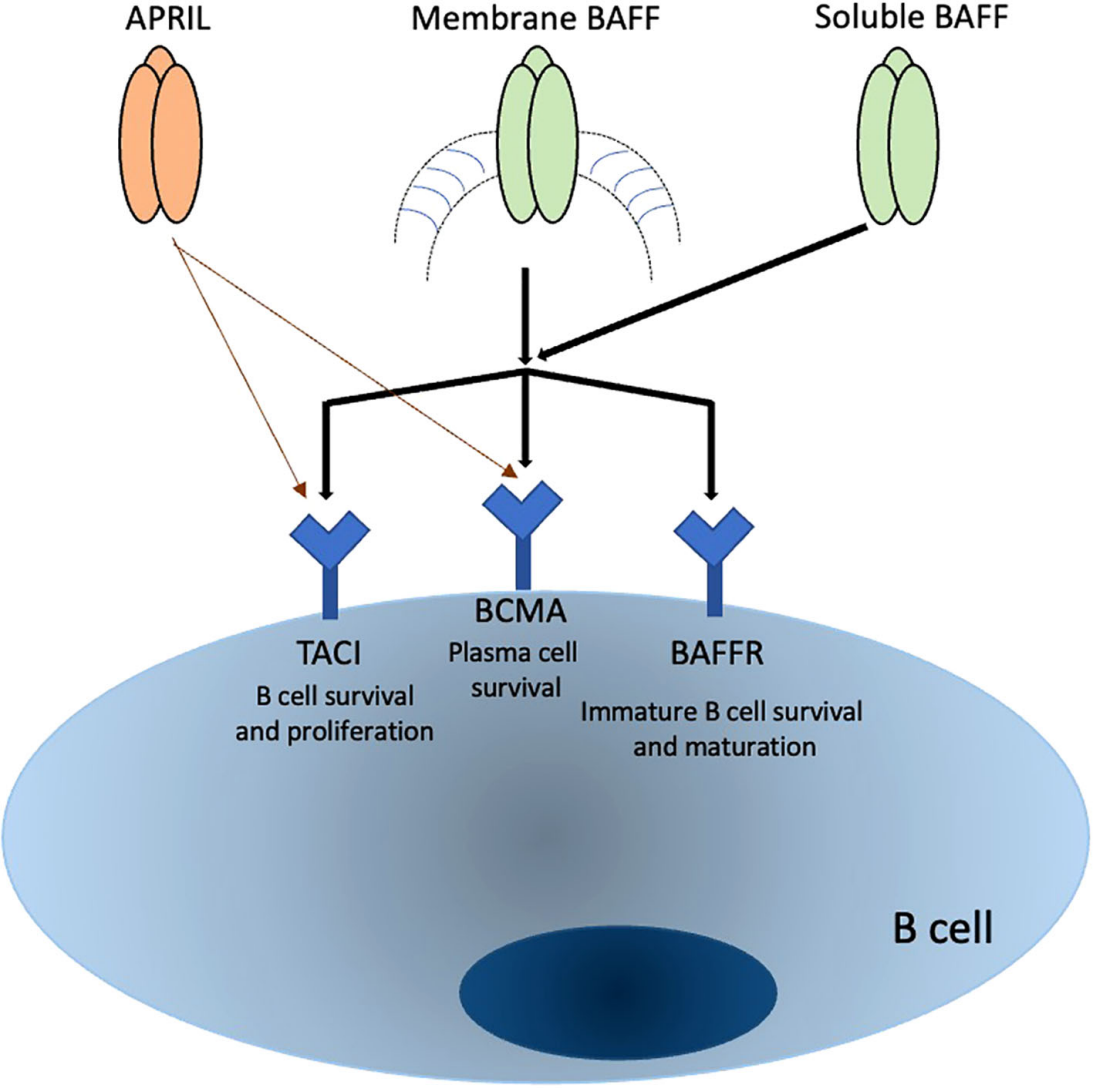
Summary of APRIL and BAFF interaction with receptors and their effects on B cells.

The expression patterns of APRIL receptors are not yet fully understood, but evidence suggests they are present on B cells at diverse levels relying on their maturation and activation status. TACI is expressed in distinct B cell populations, such as marginal zone B cells, CD27+ memory B cells, increases following B cell stimulation, and has also been identified on certain T cell subsets, especially regulatory T cells. However, this is controversial for T cells. BCMA expression is more specifically associated with differentiated B cells such as PCs, plasmablasts, and tonsillar germinal center B cells, and is critical for the survival of long-lived plasma B cells (50–52). Recent studies have shown that TACI is expressed by human memory B cells, PCs, and a subset of CD27-negative B cells. TACI expression is rapidly induced upon B cell activation, mediated through the ERK1/2 signaling pathways. BCMA expression, on the other hand, appears in memory B cells after the loss of BAFF-R expression (26, 53, 54). **Table 1** summarize the receptors associated with APRIL and the various types of white blood cells involved in autoimmune. In **Figure 1**, we summarized the interactions of both BAFF and APRIL with various receptors.

**Table 1:** provides a concise overview of how APRIL receptors interact with different white blood cell populations and highlights their functions and roles in autoimmune diseases.

Receptor	Function	Associated white blood cells
B-cell Maturation Antigen (BCMA)	Promotes survival of long-lived plasma cells (PCs)	Plasma cells (PCs), differentiated B cells
Transmembrane Activator and CAML Interactor (TACI)	Involved in B cell activation and differentiation	Memory B cells, naïve B cells, regulatory T cells (controversial)
Heparan Sulfate Proteoglycans (HSPGs)	Enhance receptor-ligand interactions	Present on various cell types including B cells, T cells

## APRIL and autoimmune disease

In recent years, the role of APRIL has emerged as crucial factors in the landscape of autoimmune diseases. This cytokine is instrumental in B cell regulation and have been implicated in various autoimmune conditions such as systemic lupus erythematosus (SLE) and rheumatoid arthritis (RA). To provide a clearer understanding of their significance, **Table 2** offers a concise overview of key aspects of APRIL in the autoimmune diseases.

**Table 2:** Role of APRIL in autoimmune diseases.

Autoimmune disease	Role of APRIL	Key findings	Implications for treatment	References
SLE	Regulates B cell survival and autoantibody production	Elevated levels of BAFF alongside conflicting APRIL measurements have been observed, with studies indicating an inverse correlation between APRIL levels and disease activity markers such as anti-dsDNA antibodies. This suggests a complex role for APRIL in modulating B cell activity and tolerance. The presence of regulatory B cells (Bregs) induced by APRIL, which produce anti-inflammatory cytokines like IL-10, indicates that APRIL may help counteract overwhelming autoreactive responses.	Given the dual role of APRIL, anti-APRIL treatments may reduce pathogenic autoantibody production and disease flares, while preserving the regulatory function of Bregs. This could mitigate disease progression and improve clinical outcomes in SLE patients.	(55–70)
RA	Contributes to B cell activity in synovial tissue	In RA, there is a significant correlation between increased APRIL levels in the synovium and inflammatory responses. APRIL is found to enhance the survival and proliferation of activated B cells in the inflamed synovial tissue. Expression of TACI and BCMA has been detected in rheumatoid synovial fibroblasts, suggesting that these cells may play an active role in local APRIL signaling, further promoting autoreactive B cell activity.	Inhibition of APRIL could lead to improved outcomes in RA by reducing the local autoantibody production and modulating B cell activity in synovial tissues. This therapeutic strategy holds promise for decreasing joint inflammation and damage.	(71–93)
SS	Limited role in inflammatory processes in salivary glands	Although serum APRIL levels are elevated in SS patients, local expression in the salivary glands is comparatively lower than BAFF. This indicates that while APRIL may play a systemic role in B cell modulation, its contribution to the inflammatory processes in the exocrine glands may be less significant. Local B cell populations in SS might rely more heavily on BAFF for their survival and function, possibly indicating a dominant role for BAFF in the pathology of SS.	The conflicting roles of APRIL suggest that further exploration is needed to understand its potential therapeutic implications in SS. Although targeted therapies might initially focus on BAFF, the role of APRIL in systemically modulating B cell dynamics warrants further investigation.	(16, 18, 23, 39–41, 79, 94–161)
IgAN	Enhances IgA-producing B cell activity	APRIL is crucial in mediating the differentiation and survival of IgA-producing B cells. Targeting APRIL has demonstrated effectiveness in reducing serum IgA levels and alleviating renal pathology in preclinical models, underscoring its pivotal role in the pathogenic processes of IgAN. It appears that APRIL facilitates class-switch recombination in B cells toward IgA, which is central to the disease mechanism.	Antagonism of APRIL could provide a novel approach to treating IgAN by inhibiting excessive IgA production and moderating the inflammatory response in the kidneys. This strategy may lead to improved renal outcomes in affected patients.	(23–25, 162–179)
Other Autoimmune Conditions	General modulation of autoreactive B cells	Increased levels of APRIL have been reported across various autoimmune conditions, yet specific mechanistic pathways remain unclear. APRIL generally promotes B cell survival and potentially contributes to autoreactivity in diverse autoimmune contexts; however, its exact role varies widely among different diseases.	Highlighting the need for individualized treatment strategies remains crucial, as the heterogeneous nature of APRIL’s impact on different B cell subsets across autoimmune diseases complicates the establishment of a uniform therapeutic approach. Tailored strategies could enhance efficacy and minimize adverse effects in patients with complex autoimmune profiles.	(1–15)

### Systemic lupus erythematosus

Systemic lupus erythematosus (SLE) is a long-term autoimmune disease that occurs more often in Caucasian people, with 3.9 cases per 100,000 women and 0.4 cases per 100,000 men. Even though treatments like corticosteroids and immunosuppressive drugs have improved, SLE still poses a significant risk of death and long-term health problems. The cause of SLE has been linked to abnormal B-cells that multiply too much (55, 56). In SLE patients, these B-cells produce too many IgG auto-antibodies, including those that target the nucleus and double-stranded DNA. The reason for lupus nephritis and eventually kidney failure can be connected with the formation of immune complexes from these autoantibodies. Because of this, targeting these overactive B-cells is a promising approach for treatment (57–59).

While it is generally agreed that high levels of BAFF in the blood of SLE patients contribute to the disease, findings about APRIL levels are less consistent. A study examining APRIL in the blood of 68 SLE patients over a median follow-up of 369 days found no link between APRIL and BAFF levels. Additionally, short-term use of corticosteroids lowered BAFF levels but did not affect APRIL levels, indicating that these two cytokines are regulated differently. Measurements of APRIL and BAFF mRNA in the blood cells of SLE patients matched their serum levels. However, it is important to be cautious when interpreting serum levels, as there is growing evidence that non-blood cells also play a significant role in producing APRIL (63–65).

Patients with positive titers of anti-dsDNA antibodies demonstrated a modest but statistically considerable reverse reference between serum levels of APRIL and anti-dsDNA antibodies. The same study also reported a moderate but important inverse correlation between APRIL serum values and disease intensity, as measured by the SLE Disease Activity Index (SLEDAI), which includes 24 descriptors (clinical, biochemical, and serological parameters, including anti-nuclear (ANA) and anti-dsDNA antibodies) with already determined serious weights (66–68). However, these inverse correlations were only observed when patients’ data were analyzed collectively, incorporating serum samples from all patients across different time points. It was suggested that APRIL may act as a downregulator of autoimmunity in SLE, although the precise mechanism was not detailed (69, 70).

Regulatory B cells (Bregs) play a crucial role in modulating immune responses via various mechanisms, but the signals required for their differentiation and activation are not yet fully understood. Research has demonstrated that overexpression of APRIL significantly reduces the incidence and severity of collagen-induced arthritis (CIA) in mice (134). Unlike BAFF, APRIL specifically promotes IL-10 production and enhances regulatory functions in human B cells (135, 136).

The hypothesis arose that APRIL could be instrumental in the induction and activation of IL-10-producing Bregs, which help suppress inflammatory responses both *in vitro* and *in vivo*. Findings illustrate that APRIL fosters the differentiation of naïve human B cells into IL-10-producing IgA+ B cells, which exhibit a Breg phenotype (136–138). These APRIL-induced IgA+ B cells successfully inhibit the activity of T cells and macrophages through the production of IL-10 and expression of PD-L1 (139, 140).

Furthermore, APRIL-induced IL-10-producing Bregs have been shown to attenuate inflammation in experimental models of autoimmune encephalitis (EAE) and contact hypersensitivity (CHS) (136, 141). Notably, a strong correlation between APRIL levels and IL-10 production has been observed in the inflamed synovial tissue of patients with inflammatory arthritis (142). Taken together, these findings underscore the potential relevance of APRIL-induced IgA+ Bregs in maintaining immune homeostasis and their implications in immunopathology.

This assumption was brought into discredit in a later study that compared APRIL serum levels among healthy individuals, patients with SLE, and patients with rheumatoid arthritis (RA). The study found that APRIL levels in the serum were significantly higher in SLE patients compared to those with RA and healthy controls. A backdated analysis indicated that APRIL serum levels inclined to be compared with anti-dsDNA antibody titers (180). Disease activity was assessed using two different indices: the British Isles Lupus Assessment Group (BILAG) index and the SLE Disease Activity Index (SLEDAI). The key distinction between these indices is that the BILAG accounts disease intensity independently across different organs, whereas the SLEDAI provides a total score for overall disease activity (181–183). In Koyama et al.’s study, serum APRIL levels did not correspond with the SLEDAI but did correspond with the BILAG index for musculoskeletal disease, particularly in cases of arthritis (184). These findings have led to the proposal of opposing therapeutic strategies.

Consistent with the findings of Stohl et al., a moderate inverse linkage was observed between APRIL serum levels and anti-dsDNA titers, with patients having higher APRIL levels showing a smaller occurrence of renal involvement (185). Additionally, the study revealed an inverse correlation between APRIL and BAFF levels in SLE patients, suggesting that APRIL and BAFF may have contrary functions in the disease. These findings support the use of specific BAFF-antagonizing agents in the treatment of SLE, as opposed to soluble TACI or BCMA receptors, which inhibit both BAFF and APRIL. However, the study by Huard et al. has shown significant increases in APRIL at both the mRNA and protein levels in bone marrow cells of NZB/W lupus mice, in contrast to control mice, while no such increases were observed in spleen cells (143). The selective blockade of APRIL using antibodies was found to delay the progression of lupus by mitigating proteinuria, kidney lesions, and mortality. This therapeutic effect was linked to a reduction in anti-DNA and anti-chromatin autoantibody levels, without disrupting the balance of B and T cell populations. Therefore, anti-APRIL treatment presents a promising alternative therapy for SLE, specifically targeting PCs with potentially fewer adverse effects compared to traditional anti-inflammatory and immunosuppressant treatments (143).

One study monitored APRIL and BAFF serum levels over 6 months in a small cohort of SLE patients (n = 10) receiving the B cell-depleting anti-CD20 antibody rituximab. CD20, which is exclusively expressed on B cells, is effectively targeted by rituximab, leading to a significant reduction in circulating B cells (186). The study found no significant distinctions in circulating APRIL levels between untreated SLE patients and controls, although BAFF levels were elevated in SLE patients. During B cell depletion, APRIL levels in the serum dropped greatly, while BAFF levels enhanced. Once B cell counts returned to normal degree, both APRIL and BAFF levels went back to their pre-treatment levels. The changes in serum levels of these two proteins during B cell depletion were quite different. The increase in serum BAFF levels observed after rituximab infusion is likely the result of two distinct mechanisms. The first is associated with the substantial reduction in receptors following B-cell depletion. The second mechanism relates to the delayed regulation of BAFF mRNA levels (144). Currently, there is no exact interpretation for these varying APRIL levels in SLE patients, but differences in the sensitivity of the ELISA tests used might be a factor.

A few studies have looked into whether genetic variations in APRIL and its receptor TACI are linked to SLE. In one study, 119 unrelated SLE patients were examined for TACI gene variations, but no significant differences from healthy controls were found, and none of the TACI mutations seemed to be related to the disease (18, 187–189). Other studies analyzed the APRIL gene in Japanese SLE patients (148 and 266 patients, respectively) and found two single-nucleotide polymorphisms (SNPs) at positions 67 (glycine/arginine) and 96 (asparagine/serine). The glycine variant at position 67 was notably more common in SLE patients. This was confirmed in a separate study, which also found that the 67 glycine + 96 asparagine combination increases risk for SLE, while the 67 arginine + 96 serine combination provides some protection. Additionally, the SNP at APRIL codon 67A was linked to SLE risk in other ethnic groups, including European-American, African-American, and Hispanic populations (190–192).

Several studies have explored the use of adenovirus-encoded or purified soluble TACI-Fc fusion proteins to block the activity of BAFF and APRIL in lupus-prone mice. Treatment with this fusion protein restrained disease progression and extended survival in these mice. One study compared the effects of TACI-Fc fusion protein with a BAFF receptor-Fc protein (which blocks only BAFF) in NZB/WF1 lupus-prone mice. Both treatments were similarly effective in these mice (193, 194). However, TACI-Fc treatment led to lower IgM serum levels, a reduction in splenic PCs, and a diminished IgM response to a T cell-dependent antigen. This was likely due to APRIL’s influence on the production of neutralizing anti-IgM antibodies by B cells. Therefore, a specific BAFF-blocking agent might be more advantageous, as agents blocking both BAFF and APRIL could disrupt B cell production of neutralizing anti-IgM antibodies, potentially increasing susceptibility to infections (24, 195, 196).

Lupus nephritis (LN) is a significant manifestation of SLE, where the interplay of BAFF (B-cell activating factor) and APRIL (a proliferation-inducing ligand) plays a crucial role in the disease’s pathogenesis (151). In patients with LN, elevated levels of these cytokines can indicate renal inflammation and tissue damage. While BAFF overexpression has been linked to the activation of B-cells and the development of autoimmune responses, its presence in the kidneys—especially in class III and IV LN—is noteworthy, as it correlates strongly with kidney activity indices (152, 153). On the other hand, APRIL’s expression is also heightened in the renal compartments of patients with proliferative LN, suggesting its role in exacerbating autoimmune inflammation and promoting the influx of macrophages (151, 154). Together, the detection of BAFF and APRIL, alongside their receptor interactions in urinary samples, highlights their potential as non-invasive biomarkers for monitoring disease progression and therapeutic response in LN, providing hope for more personalized and effective management of SLE patients. This emphasizes the need for further research to solidify their roles in clinical settings, paving the way for advancements in treating this complex condition.

### Rheumatoid arthritis

Arthritis is an inflammation affecting one or more joints, leading to symptoms such as swelling, pain, and limited movement. While there are many types of inflammatory arthritis, rheumatoid arthritis (RA) is the most prevalent. RA primarily impacts the synovium, the tissue lining the joints, which is usually a thin layer with one or two cell layers deep. In RA, this synovial tissue undergoes hyperproliferation of fibroblast-like synoviocytes (FLS) in the lining layer and experiences infiltration by macrophages and T and B cells in the sub-lining. This infiltration contributes to inflammation and causes damage to the bone and cartilage. RA is also marked by the generation of auto-antibodies (71–73).

The connection between APRIL and rheumatoid arthritis (RA) was initially suggested by mouse researches. In these studies, mice susceptible to arthritis were immunized with type II collagen (CII) from other species, resulting in a disease model similar to human RA known as collagen-induced arthritis (CIA). Both CII antibodies and CD4+ T cells are necessary for developing CIA. Using TACI-Fc to inhibit APRIL and BAFF in this model prevented disease progression and reduced disease severity compared to controls (74–77).

Further research showed that synovial fluid from patients with inflammatory arthritis had significantly higher levels of APRIL compared to those with non-inflammatory arthritis like osteoarthritis. Although BAFF levels were also higher in patients with inflammatory arthritis, they did not correlate with APRIL levels. This suggests that both cytokines may play different roles in managing pathogenic B or T cells in inflamed joints (78–81). Curiously, a study investigating serum from patients with various systemic immune diseases (including SLE, RA, Reiter’s syndrome, psoriatic arthritis, polymyositis, and ankylosing spondylitis) found elevated APRIL levels and the presence of APRIL/BAFF hetero-trimers. Given the limited sample size, it will be important to investigate whether APRIL/BAFF hetero-trimers are more prevalent in specific rheumatic diseases and if they have functional significance (82–84).

Another study explored serum from 16 RA patients and found elevated levels of APRIL compared to controls. Importantly, they discovered that synovial fibroblasts in RA patients express BCMA, whereas this expression was not observed in cells from patients with osteoarthritis. APRIL therapy was shown to increase cell proliferation and stimulate the production of pro-inflammatory cytokines like IL-6 and TNF-alpha, indicating that APRIL plays a key role in the development of RA (85).

Additional evidence of APRIL’s involvement in RA comes from two investigations. In one study, APRIL and BAFF serum levels were monitored over 6 months in nine RA patients treated with rituximab. APRIL levels were elevated in these RA patients compared to controls, while BAFF levels were not. During B cell depletion, APRIL levels remained stable, whereas BAFF levels raised substantial, similar to what was observed in SLE patients (86, 87).

APRIL’s role in RA was further highlighted by a study examining synovial biopsies from 72 RA patients, focusing on B cell function and the expression of APRIL and BAFF. The synovitis, or inflamed synovial tissues, were categorized basing on their lymphoid structure into: ectopic germinal centers (GCs), T cell–B cell aggregates without germinal center reactions, and disorganized diffuse infiltrates (88–90). About half of the RA patients had synovitis with GCs or T cell–B cell conglomerations, while the other half had dispersed infiltrates. APRIL was exposed to be particularly expressed in CD83+ dendritic cells, with the maximum expression in GC synovitis, while BAFF was similarly expressed across different types of synovitis and localized to CD68+ macrophages. CD138+ PCs and some T cells in aggregate and diffuse synovitis expressed TACI, but not in GC synovitis (91, 92).

To investigate functional differences between these compartments, synovium-SCID mouse chimeras were cured with TACI-Fc. This treatment led to the loss of GCs in the synovial tissue, reduced Ig production, and lower IFN-gamma production. In contrast, in aggregate and diffuse synovitis, TACI-Fc treatment had no effect on Ig levels but increased IFN-gamma production. These findings suggest that TACI ligands may have either stimulatory or inhibitory effects depending on the type of synovitis (93, 197).

In rheumatoid arthritis (RA), the synovial tissue becomes a hub of autoreactive B-cell activity driven by the expression of key cytokines BAFF and APRIL (79, 145). Synovial fibroblasts (RASF) play a pivotal role in this process, as they constitutively release high levels of both BAFF and APRIL, thereby supporting the activation, proliferation, and differentiation of B cells (146). The presence of ectopic lymphoid structures (ELSs) within the RA synovium facilitates crucial processes such as clonal expansion and immunoglobulin class-switching, while the expression of activation-induced cytidine deaminase (AID) is essential for these events to occur (147, 148). Excitingly, RASF have been shown to enhance the expression of AID and the production of class-switched antibodies, primarily IgG, particularly following Toll-like receptor 3 (TLR3) stimulation. This interaction not only fosters the ongoing production of autoantibodies but also indicates a complex interplay between RASF and B cells, highlighting the significance of BAFF/APRIL signaling in enabling these immune responses (149, 150). The nuanced understanding of how these factors contribute to B-cell activation and differentiation in the rheumatoid synovium opens avenues for potential therapeutic interventions that target these pathways, aiming to mitigate the autoimmune processes inherent in RA.

### Sjögren’s syndrome

Sjögren’s syndrome (SS) is an autoimmune condition influencing exocrine glands and often occurs alongside RA and SLE. Patients with SS commonly have anti-nuclear antibodies SSA/Ro and SSB/La, with SSB/La being more specific to SS. Symptoms similar to SS were observed in BAFF transgenic mice, but not in APRIL transgenic mice (94–96). Despite this, elevated serum levels of both APRIL and BAFF have been found in SS patients, especially those positive for SSA/Ro. There is also a positive connection between the serum levels of these two cytokines, though their exact role in SS is still not fully understood (97, 98).

In patients with Sjögren’s syndrome (SS), the expression of BAFF (B-cell activating factor) and APRIL (a proliferation-inducing ligand) in the salivary glands reveals important insights into the complex immune landscape of this condition (155). Epithelial cells within the minor salivary glands are known to be robust producers of BAFF, playing an active role in antigen presentation and modulating immune responses. However, while BAFF levels remain relatively stable between SS patients and healthy individuals, the expression of APRIL is less prominent and predominantly localized to the ductal epithelial cells, showing a decrease in SS patients compared to healthy individuals (156, 157). This suggests that APRIL may not play a central role in the inflammatory processes within the salivary glands, especially in comparison to its counterpart, BAFF (158). Interestingly, elevated levels of APRIL have been detected in the serum of SS patients, particularly among those who are anti-Ro/La positive, raising questions about the origins of serum APRIL and its relationship to salivary gland dysfunction (159, 160). The association of serum APRIL with decreased salivary flow implies a complex interplay between systemic inflammatory signals and local glandular activity. Furthermore, TACI expression in the salivary glands may reflect intricate interactions between BAFF, APRIL, and their receptors in maintaining homeostasis or potentially exacerbating dysfunction in SS (161). Overall, while BAFF stands out as a significant factor in modulating B-cell activity and influencing inflammation in SS, APRIL appears to have a subtler role, warranting further investigation to clarify its function and therapeutic potential in managing salivary gland involvement in this autoimmune condition.

### IgA nephropathy

IgA nephropathy (IgAN) arises from the deposition of immunoglobulin A (IgA) in the glomeruli, leading to kidney damage and varying degrees of proteinuria. One of the innovative treatments targeting this condition is telitacicept, which acts on critical signaling molecules BAFF and APRIL to modulate B cell development. Clinical trials have shown that telitacicept can significantly reduce proteinuria while exhibiting a favorable safety profile (162, 163). Its dual-target approach distinguishes it from other B cell-related therapies (164). This unique mechanism underscores telitacicept’s potential in treating IgAN and encourages further exploration of novel therapeutic strategies in this field (165). By leveraging agents like telitacicept, we can expand our treatment options and enhance patient outcomes in autoimmune conditions.

In a phase II study known as the JANUS trial (NCT02808429), researchers evaluated atacicept, a B-cell–targeted immunomodulator, in patients with IgAN experiencing persistent proteinuria (≥1 g/d or 0.75 mg/mg on 24-hour urine protein-to-creatinine ratio) despite optimal standard care. Sixteen patients were randomized to receive weekly subcutaneous injections of either placebo (n = 5), atacicept 25 mg (n = 6), or atacicept 75 mg (n = 5) (179). The trial revealed an acceptable safety profile, with 75% of patients completing at least 48 weeks of treatment and 50% completing 72 weeks, followed by a 24-week safety follow-up. Treatment-emergent adverse events (TEAEs) were reported in 14 patients, most of which were classified as mild or moderate, and only three serious TEAEs occurred, none of which were related to the treatment. Atacicept led to significant, dose-dependent reductions in levels of IgA, IgG, IgM, and galactose-deficient IgA1 (Gd-IgA1) at week 24, effects that were sustained up to week 72. Additionally, early reductions in proteinuria were observed at week 24, and while renal function declined in the placebo group, it remained stable in patients receiving atacicept. These findings suggest atacicept’s effectiveness in reducing levels of pathogenic Gd-IgA1 and its potential benefits for proteinuria and renal function in patients with IgAN (179).

Another promising agent, sibeprenlimab, was investigated in the phase 2 ENVISION trial (NCT04287985). This humanized IgG2 monoclonal antibody neutralizes APRIL and was tested in adults with biopsy-confirmed IgAN at high risk for disease progression. Participants were randomized in a 1:1:1:1 ratio to receive either sibeprenlimab at doses of 2 mg/kg, 4 mg/kg, or 8 mg/kg, or placebo, administered intravenously once monthly for 12 months. The primary endpoint measured changes from baseline in the log-transformed 24-hour urinary protein-to-creatinine ratio at month 12, with secondary endpoints including changes in estimated glomerular filtration rate (eGFR) (25). Among the 155 randomized patients, significant reductions in the urinary protein-to-creatinine ratio were noted at 12 months, with geometric mean ratio reductions of 47.2% (2 mg/kg), 58.8% (4 mg/kg), and 62.0% (8 mg/kg), versus 20.0% in the placebo group. EGF changes from baseline at 12 months were -2.7 ml/min/1.73 m² (2 mg/kg), 0.2 ml/min/1.73 m² (4 mg/kg), -1.5 ml/min/1.73 m² (8 mg/kg), and -7.4 ml/min/1.73 m² (placebo). Adverse events occurred in 78.6% of the sibeprenlimab groups and 71.1% in the placebo group, indicating a comparable safety profile. Sibeprenlimab treatment significantly decreased proteinuria compared to placebo, suggesting its potential as a therapeutic option for patients with IgA nephropathy.

Investigations into the efficacy of systemic corticosteroids for treating IgAN have yielded mixed results. Two major studies, STOP-IgAN (174) and TESTING (175), showed that corticosteroids did not significantly improve kidney function or reduce the decline in eGFR, and were associated with numerous adverse events, prompting the early termination of the TESTING trial. In contrast, emerging therapies that target APRIL present an encouraging alternative; by modulating the immune response and decreasing pathogenic IgA production, these treatments may offer both safety and efficacy, improving patient outcomes in IgAN management. Continued research into the role of APRIL in tailoring therapies for IgAN may represent a significant advance in renal immunology. In **Table 3**, we summarize the main therapeutic agents and their features.

**Table 3:** Comparison of therapeutic agents targeting APRIL and BAFF.

Therapeutic agent	Mechanism of action	Disease	Current development stage	References
Atacicept	Dual inhibition of BAFF and APRIL	RA, SLE	Tested in clinical trials	(19–21, 174, 175)
Rituximab	Anti-CD20 antibody; depletes B cells	RA, SLE	Approved and used off-label	(57–59, 115)
Belimumab	BAFF antagonist; inhibits B cell survival	SLE	Approved for SLE	(60–62)
Telitacicept (RC18)	Targets both BAFF and APRIL; enhances B cell modulation	IgAN, RA, SLE	Phase 1 trials	(198, 199)
Anti-APRIL monoclonal antibodies	Specifically target APRIL, depleting TACI/BCMA+ B cells	Potential in multiple autoimmune diseases	Preclinical studies ongoing	(23, 100, 170)

## Synergistic targeting both APRIL and BAFF

Simultaneously targeting APRIL and BAFF has shown promise in achieving synergistic therapeutic outcomes in certain autoimmune conditions, as evidenced by early clinical data and preclinical models. Both Atacicept and Telitacicept are notable examples of therapies that target these two critical cytokines, which play vital roles in B cell survival and differentiation. In phase II clinical trials, Atacicept demonstrated a dose-dependent reduction in flare rates among patients with SLE, indicating that inhibiting both BAFF and APRIL can lead to improved clinical outcomes (200). Similarly, Telitacicept has shown robust efficacy and safety in patients with SLE and has successfully met endpoints in clinical trials, highlighting the potential benefits of dual inhibition. The observed benefits may stem from a more comprehensive modulation of B cell activity, potentially addressing both hyperactivity and autoreactive tendencies that characterizes various autoimmune diseases (201).

The rationale for simultaneously targeting BAFF and APRIL lies in their shared but distinct mechanisms. BAFF promotes B cell survival and maturation, while APRIL supports the differentiation of plasma cells, which are the cells responsible for producing antibodies, including autoantibodies. By inhibiting both pathways, these therapies may more effectively reduce the pool of autoreactive B cells and plasma cells compared to targeting either cytokine alone (202). Evidence suggests that excessive BAFF levels post-B cell depletion, such as after treatments like rituximab, can lead to the re-emergence of autoreactive B cells, which implies that sustained inhibition of both BAFF and APRIL during treatment could mitigate this risk.

Several autoimmune conditions may particularly benefit from this dual-targeting approach. For instance, conditions like systemic lupus erythematosus and Sjögren’s syndrome, where abnormal B cell proliferation and autoantibody production are core features, could see improved patient outcomes through combined BAFF and APRIL inhibition (203). Moreover, conditions like rheumatoid arthritis and systemic sclerosis, which also involve complex B cell dysregulation, may be amenable to this synergistic strategy. Additionally, diseases characterized by fluctuating levels of BAFF, such as bullous pemphigoid and pemphigus vulgaris, might benefit from well-timed combinations of these therapies to prevent the rebound of pathogenic B cells after depletion (204).

In conclusion, the strategy of simultaneous targeting of APRIL and BAFF represents a promising avenue in the treatment of autoimmune disorders. The existing preclinical and early-phase clinical data provide a strong foundation for further exploration into this combined approach, particularly in conditions marked by significant B cell involvement. As more clinical trials progress, optimizing the timing and dosing of these dual-targeted therapies could herald a new era of effective treatments for autoimmune diseases. Notably, the precise identification of patient populations that would benefit most from this strategy remains a crucial component of future research efforts.

## Comparison with other B-cell targeting strategies

### Targeting Bruton’s tyrosine kinase

BAFF and APRIL targeted therapies differ significantly from BTK inhibitors like ibrutinib in several key aspects, including their mechanisms of action, clinical efficacy, and safety profiles.

BAFF and APRIL play crucial roles in B cell survival and differentiation. Therapies targeting these cytokines, such as belimumab or anti-APRIL antibodies, primarily aim to modulate B cell activity by inhibiting their overactivity, which is a hallmark of many autoimmune diseases (205). In contrast, BTK inhibitors block the signaling pathways activated by B cell receptors (BCRs), affecting downstream processes that impact B cell development and immune responses. BTK inhibition disrupts both B cell and myeloid lineage differentiation, impacting inflammation indirectly. This means that while BAFF and APRIL therapies directly affect B cells’ survival and proliferation, BTK inhibitors have a broader mechanism that also affects the inflammatory milieu generated by these cells (206, 207).

Clinical trials for BTK inhibitors have shown variable success in treating autoimmune conditions like RA, with some early-stage studies indicating modest improvement after prolonged periods (e.g., fenebrutinib’s effects becoming significant at 12 weeks). However, the clinical success rate for BTK inhibitors in RA has been relatively poor compared to the promising results of BAFF and APRIL-targeted therapies, which have generally shown better efficacy across multiple autoimmune diseases (208). For instance, in SLE and Sjögren’s syndrome, BTK inhibitors have not demonstrated the expected therapeutic benefit despite effective action in preclinical models. This highlights a notable disconnect in efficacy when transitioning from animal models to human clinical contexts for BTK inhibitors (209).

The safety profiles of these two therapeutic classes also differ. BAFF and APRIL-targeted therapies are generally well-tolerated, with predictable side effects related to reduced B cell activity, such as increased susceptibility to infections. Conversely, BTK inhibitors can have more complex safety issues, including potential impacts on both B cells and myeloid lineage cells, which could lead to varied inflammatory responses or opportunistic infections. The prolonged nature of treatment with BTK inhibitors necessitates careful monitoring, especially due to the cumulative effects on B cell and myeloid lineage differentiation, which may take time to manifest (210).

Furthermore, the therapeutic impact of BTK inhibitors may necessitate a precision medicine approach, where patient populations are stratified based on specific immune signatures. This is in contrast to BAFF and APRIL therapies, which have more broadly applicable mechanisms targeting B cell dysregulation without the need for complex patient matching based on underlying immunological profiles (211).

Overall, while both BAFF/APRIL-targeted therapies and BTK inhibitors aim to modulate dysfunctional B cell responses in autoimmune diseases, they operate via distinct mechanisms, exhibit varying clinical efficacies and safety profiles, and may require different approaches in patient management and precision medicine. This divergence underscores the importance of understanding the underlying biological mechanisms when selecting therapeutic strategies for patients with autoimmune conditions (87).

### Anti-CD19 monoclonal antibodies

BAFF and APRIL targeted therapies differ significantly from anti-CD19 monoclonal antibody therapies in several fundamental aspects, including their mechanisms of action, clinical efficacy, and safety profiles (212).

BAFF and APRIL therapies primarily modulate B cell survival and differentiation by blocking these critical cytokines that support B cell maturation and survival. By inhibiting BAFF and APRIL, these treatments reduce the number of autoreactive B cells and decrease overall B cell hyperactivity, which is a significant factor in various autoimmune diseases. In contrast, anti-CD19 monoclonal antibodies directly target the CD19 molecule on the surface of B cells, affecting all stages of B cell development, including pre-plasmablasts and plasma cells (23, 87). This broad targeting means that anti-CD19 therapies may have a more immediate and profound effect on B cell populations, potentially depleting both autoreactive and non-autoreactive B cells.

The clinical efficacy of BAFF and APRIL-targeted therapies has shown promise across different autoimmune diseases, with evidence suggesting improvements in disease states like SLE. On the other hand, the clinical outcomes for anti-CD19 therapies have been less encouraging. For example, trials involving Obexelimab, an anti-CD19 antibody, were halted after failing to meet predefined endpoints in SLE patients and also showed limited data on efficacy in RA. This contrasts starkly with the more consistent positive results observed with BAFF and APRIL antagonists, which have been linked to better management of symptoms in several autoimmune contexts (213, 214).

In terms of safety, BAFF and APRIL-targeted therapies tend to have manageable safety profiles, primarily associated with their role in modulating B cell activity without completely eliminating B cell populations. This results in reduced risk for severe infections compared to therapies that lead to total or nearly total B cell depletion. Conversely, anti-CD19 monoclonal antibodies could lead to a more significant reduction in B cell numbers, increasing the risk of infections and related complications. Additionally, patients treated with anti-CD19 therapies may face risks related to the clearing of entire B cell populations, including potential complications from the depletion of protective immune functions (215).

The target populations for these therapies also differ. BAFF and APRIL antagonists may be more favorable for patients with a certain profile of B cell dysregulation that is responsive to such cytokine blockade. In contrast, anti-CD19 therapies might be more appropriate for conditions where a broader B cell depletion could be beneficial, although their efficacy remains uncertain. Moreover, the presence of CD19high B cells linked to autoimmunity and poor outcomes after treatment brings into question the overall benefit of depleting CD19-expressing cells in certain populations (87).

Overall, while both BAFF/APRIL-targeted therapies and anti-CD19 monoclonal antibodies aim to modulate B cell function in autoimmune diseases, they do so through different mechanisms that result in varying clinical efficacies and safety profiles. The former tends to have a more favorable track record in clinical outcomes and manageable side effects, contrasted by the challenges faced by anti-CD19 therapies in achieving predefined efficacy endpoints, raising important considerations for clinical practice and the selection of therapeutic strategies for patients with autoimmune disorders.

## Safety concerns and potential adverse effects of APRIL targeting

While APRIL-targeted therapies show promise in managing autoimmune conditions, several potential adverse effects warrant careful consideration. One significant concern is the increased risk of infections. Since APRIL plays a crucial role in B cell survival and differentiation into antibody-producing plasma cells, its inhibition could impair the body’s ability to mount effective immune responses against pathogens (216). Clinical observations have indicated that therapies like Atacicept, which target both APRIL and BAFF, are associated with higher rates of infections—especially respiratory infections—compared to placebo groups. This elevation in infection risk underscores the need for vigilant monitoring in patients undergoing such treatments (217).

Another concern surrounding APRIL-targeted therapies is the diminished responsiveness to vaccinations. Vaccinations rely on the body’s ability to generate robust antibody responses, which may be compromised through the depletion of plasma cells that produce these antibodies. In clinical settings, patients on therapies that influence B cell dynamics have demonstrated reduced antibody responses to vaccines, such as those for influenza and pneumococcus. A study involving patients treated with Atacicept found that the antibody response to vaccination was notably attenuated, prompting recommendations for careful consideration of vaccination schedules before initiating APRIL-targeted therapies (218).

Long-term depletion of plasma cells is another potential adverse effect. Plasma cells are essential for maintaining long-term antibody production, especially for combating recurrent infections. If APRIL activity is chronically inhibited, it may lead to insufficient plasma cell levels and, consequently, a long-term inability to produce adequate protective antibodies. Evidence from preclinical models indicates that prolonged targeting of APRIL can lead to lasting reductions in plasma cell populations, raising concerns about the durability of humoral immunity beyond the immediate scope of treatment (107, 219).

Moreover, the emerging understanding of the role of APRIL in maintaining immune tolerance has also raised alarms regarding the potential for increased autoimmunity. In some cases, targeting APRIL may inadvertently lead to an imbalance in the immune system, allowing for the proliferation of autoreactive B cells. For instance, research indicates that while reducing APRIL can decrease certain autoantibody levels, it might also disrupt the regulatory pathways that prevent autoimmunity, leading to unexpected adverse immune reactivity (220).

Finally, it is essential to acknowledge that while ongoing and future clinical trials are expected to provide a clearer picture of these risks, data from existing studies illustrate the complex balance between therapeutic efficacy and safety in APRIL-targeted therapies. As with any treatment modality, personalized approaches considering individual patient profiles and associated risks are critical in the management of autoimmune diseases.

Exploring potential resistance mechanisms to APRIL-targeted therapies is essential for understanding their long-term efficacy. We can list the several key areas of concern:

1. Compensatory Upregulation of BAFF Signaling: One major resistance mechanism is the compensatory upregulation of BAFF (B-cell-activating factor). Since BAFF and APRIL share overlapping functions in supporting B cell survival, inhibition of APRIL may lead to increased BAFF levels as the immune system attempts to compensate for the decreased signals. Studies have indicated that when APRIL is inhibited, BAFF can facilitate the survival of autoreactive B cells, which may eventually undermine the treatment’s effectiveness. This finding suggests that therapies targeting APRIL may need to simultaneously consider BAFF modulation to maintain balance (30).
2. Alternative Survival Pathways: Besides BAFF, B cells may utilize other survival pathways when APRIL signaling is disrupted. For instance, upregulation of different cytokines that promote B cell survival could occur in response to APRIL blockade. Investigating these alternative pathways is crucial, as they can provide insights into how B cells adapt and survive despite APRIL-targeted therapy. It may become necessary to target multiple pathways to achieve a more durable therapeutic effect (221).
3. Subpopulation Resilience: Different subpopulations of B cells might exhibit varying resilience to APRIL blockade. For instance, memory B cells and certain activated B cell subsets may be less susceptible to loss of APRIL signaling. Understanding the heterogeneity within B cell populations can help in predicting how some cells might resist therapy and how they could contribute to disease persistence or relapse (222).
4. Potential for Autoimmunity: As mentioned earlier, the disturbance of APRIL signaling can lead to an imbalance in immune regulation, promoting autoimmunity rather than treating it. This potential for autoimmunity should be closely monitored as a form of resistance, as the emergence of new autoreactive B cells could overshadow the therapeutic benefits of APRIL blockade (87).
5. Pharmacodynamics and Dosing Considerations: The long-term use of APRIL-targeted therapies may lead to pharmacodynamic changes where the initial efficacy diminishes over time. Strategies to optimize dosing regimens and treatment durations are vital to maintaining therapeutic benefits without encouraging resistance mechanisms (223).

## Expectations from APRIL targeting

The exploration of B-cell modulatory agents has shown considerable promise in treating autoimmune diseases. Rituximab, an anti-CD20 antibody, was one of the first successes, followed by belimumab, a BAFF antagonist that restricts the survival of mature B cells. Atacicept, which inhibits both BAFF and APRIL, has also emerged as a noteworthy treatment option. Recently, telitacicept (RC18), an advanced version of TACI-Fc with a higher molecular weight, has been tested in Phase 1 trials for rheumatoid arthritis and lupus, showing effects on circulating B-cell counts and immunoglobulin levels similar to atacicept (18, 99, 198, 199).

Despite the absence of human trials specifically targeting APRIL alone, preclinical studies have validated the efficacy of monoclonal antibodies that selectively target human APRIL. These studies indicate potential effectiveness in conditions like multiple myeloma, with no significant barriers identified for applying these treatments in autoimmune diseases (23, 100–103). The crucial role of APRIL in plasma cell (PC) regulation is well-documented; however, its influence on the early stages of the humoral response remains less clear compared to anti-CD20 and anti-BAFF therapies. Notably, while clinical trials have been unable to investigate the depletion of bone marrow PCs due to the invasiveness of required procedures, findings from mouse models suggest that CD20 expression is absent in PCs, indicating that existing CD20-targeted therapies may not significantly impact these long-lived cells (104–108).

Interestingly, BAFF receptor expression patterns mirror those of CD20, with complete loss at the PC differentiation stage, highlighting its critical role in B cell development and survival. Unlike BCMA, which is present on all PCs but does not respond to BAFF, TACI has been shown to respond to a highly oligomerized form of BAFF, suggesting that PCs largely operate independently of BAFF signaling (109–113). This independence might extend to humans, as evidenced by belimumab’s limited effect on protective immunity from long-lived PCs produced after vaccination (114, 115). However, evidence from recent studies, such as that by Eslami et al., suggests that the TACI/BAFF axis may still play a significant role in PC survival (24).

In terms of treatment outcomes, belimumab effectively reduces IgM production but not IgG, while anti-APRIL strategies are gaining traction due to their impact on PC survival and antibody production, as shown in animal studies (116, 117). The current landscape of B-cell modulation reveals that no agent conclusively impacts long-lived BM PCs in humans, underscoring the potential for APRIL antagonists to fill this therapeutic gap (118, 119).

Povetacicept (ALPN-303), a high-affinity antagonist of both APRIL and BAFF, has been shown to significantly inhibit B cell proliferation and antibody secretion more effectively than existing alternatives, including anti-CD20 monoclonal antibodies (23). In various mouse models, this agent reduced serum immunoglobulin levels and improved outcomes in lupus nephritis, suggesting substantial potential for the treatment of autoantibody-mediated autoimmune diseases.

Moreover, trials such as ILLUMINATE have evaluated tabalumab, displaying biological activity consistent with BAFF inhibition, yet failing to demonstrate significant clinical efficacy in treating SLE (178). Atacicept’s long-term efficacy has also been documented, highlighting its ability to sustain beneficial outcomes in IgA nephropathy (178).

As research progresses, the exploration of APRIL antagonism may yield important insights and innovative therapeutic strategies, particularly for diseases driven by pathogenic antibodies from long-lived PCs, warranting further investigation and clinical trials (19, 133).

Long-term inhibition of APRIL can significantly impact protective antibody responses, particularly concerning vaccine-induced immunity and the maintenance of memory plasma cells. APRIL plays a crucial role in the survival and differentiation of B cells into antibody-producing plasma cells. If APRIL signaling is inhibited over an extended period, it could compromise the ability of B cells to respond effectively to vaccines, which may lead to reduced vaccine efficacy. Without adequate APRIL signaling, there could also be challenges in class switch recombination, preventing B cells from producing different classes of antibodies, ultimately resulting in suboptimal immune responses (224).

In terms of memory plasma cell maintenance, APRIL is vital for developing and sustaining long-lived memory B cells. Prolonged APRIL inhibition may hinder the formation of these cells after vaccination, diminishing the body’s ability to mount quick and robust responses upon re-exposure to pathogens. Memory plasma cells, which are crucial for long-term immunity through continuous antibody production, could also suffer from inadequate survival signals, leading to reduced serum antibody levels and waning immunity (225).

To mitigate these concerns, strategies can be implemented. One approach involves utilizing intermittent therapy, where APRIL inhibition is not continuous but rather given in cycles. This allows periods where normal APRIL signaling can occur, supporting memory B cells and plasma cell maintenance during and after vaccinations. Combination therapies could also be effective. For instance, administering BAFF along with APRIL inhibitors might maintain B cell survival and function, balancing the effects on immunity (226).

Vaccine design can be optimized by incorporating adjuvants that stimulate multiple immune pathways, enhancing B cell activation without solely relying on APRIL signaling. Implementing regular monitoring of antibody levels and memory B cell populations in patients receiving long-term APRIL inhibition can provide insights into waning immunity, allowing for timely adjustments in therapy. For example, temporarily resuming APRIL signaling through BAFF administration when necessary could help maintain protective immunity (227, 228).

Moreover, research into small molecules or biologics that specifically promote the formation and longevity of memory B cells, independent of APRIL, might provide additional pathways to strengthen vaccine responses in patients undergoing APRIL inhibition. Overall, while the challenges posed by long-term APRIL inhibition are significant, a thoughtful and multifaceted approach can help ensure that protective antibody responses and memory plasma cell maintenance are preserved, ultimately benefiting patient outcomes (100).

## Future directions

Moving forward, research should prioritize the development of personalized treatment strategies that identify specific patient populations likely to benefit from APRIL-targeted therapies. Utilizing biomarkers and immune profiling can enhance our ability to tailor interventions to the unique immune landscapes of individual patients, optimizing therapeutic outcomes. Additionally, it is essential to investigate the long-term implications of APRIL inhibition, particularly regarding its effects on vaccine responses and the maintenance of memory B cells. Understanding how prolonged inhibition might impact humoral immunity will be key in guiding treatment plans.

Moreover, exploring dual-target strategies that simultaneously inhibit both APRIL and BAFF may yield synergistic effects, offering a more comprehensive approach to modulating B cell activity in autoimmune diseases. Given the distinct functions of these cytokines, such combined therapies could address the complexities inherent in disorders marked by B cell dysregulation more effectively than targeting either pathway alone.

## Conclusion

This review highlights the intricate relationship between APRIL and autoimmune diseases, revealing the therapeutic potential and limitations of targeting this cytokine. APRIL plays a significant role in B cell regulation and pathogenic autoantibody production, making it a critical player in conditions such as systemic lupus erythematosus, rheumatoid arthritis, and Sjögren’s syndrome. While targeting APRIL presents promising opportunities for improving disease management, caution is warranted due to its dual role in immune regulation. The emerging evidence from clinical trials, particularly in IgA nephropathy, underscores APRIL’s promise as a therapeutic target.

As research progresses, a deeper understanding of APRIL’s functions across various autoimmune contexts will be essential for developing tailored therapies that maximize efficacy while minimizing adverse effects. By harnessing the specificity of APRIL-related interventions, the field stands to make significant strides in the effective treatment of autoimmune diseases, paving the way for innovative strategies that address the complexities of these conditions.
